# The use of non-invasive ventilation by emergency doctors in Johannesburg Academic Hospitals, South Africa – assessing knowledge, attitudes and practices

**DOI:** 10.1016/j.afjem.2023.11.002

**Published:** 2023-11-11

**Authors:** Dr Holly Bird, Dr Craig Beringer, Dr Pano Parris

**Affiliations:** Division of Emergency Medicine, Faculty of Health Sciences, School of Clinical Medicine, University of the Witwatersrand, Johannesburg, South Africa

**Keywords:** Non-invasive ventilation, Knowledge, Attitude, Emergency doctor, Training

## Abstract

**Introduction:**

Non-invasive ventilation (NIV) is an alternative ventilatory method to endotracheal intubation and invasive ventilation in patients presenting in acute respiratory failure. Appropriate and timely application of NIV has shown benefits over invasive ventilation. In recent years, there has been an increase in the use of NIV for varying pathologies in response to limited resources.

Emergency department doctors’ knowledge of NIV, as well as their attitude towards its use can have significant effects on the success of NIV. The aim of this study was to assess emergency doctors’ use of NIV in the South African setting.

**Methods:**

This was a multi-centre prospective cross-sectional study that was conducted across three academic emergency departments in Johannesburg, South Africa. Doctors of various grades were included in the study and their responses were analysed according to their level of experience and their job description.

**Results:**

The mean knowledge score of the participants was shown to increase with an increase in job designation (*p* < 0.001). The doctors’ attitude towards NIV was more positive in those with higher knowledge scores (*p* < 0.001). Participants with previous critical care experience versus those without was associated with a higher average knowledge score (77% vs. 69 %, *p* = 0.009) as was formal NIV training versus those without (77% vs. 69 %, *p* = 0.01). The COVID-19 pandemic increased confidence in the use of NIV in 61 % of participants. The majority (69 %) of participants did not use a checklist when administering NIV. Only 53 % used a guideline when initiating a patient on NIV and just 26 % used a locally produced protocol.

**Conclusion:**

Increased clinical experience, critical care exposure and formal NIV training corresponded with higher knowledge scores. Formal NIV training programs, the implementation of a checklist and the development of locally produced protocols are recommended to improve knowledge, attitudes and NIV practice that is in-keeping with international standards.

## African relevance

• Working in a resource-limited setting necessitates the need for reliable, alternative ventilation strategies.
• The limited availability of Emergency Medicine Specialists often leads to non-specialists and junior doctors initiating NIV.
• Formal NIV training should be developed with junior doctors in mind and in-keeping with international standards.

## Introduction

Non-invasive ventilation (NIV) is a method of providing ventilatory support to a patient in acute respiratory failure via a face mask or similar device [1]. The role of NIV in the emergency department (ED) is well established, with a robust evidence base for its use in certain medical conditions [[1], [2], [3], [4], [5], [6], [7]]. Resource limitations such as those experienced in low to middle income countries as well as the advent of the COVID-19 pandemic have led to the use of NIV as an alternative ventilatory measure to invasive ventilation [[8], [9], [10], [11], [12], [13]].

The decision to utilise NIV is often made in the ED, where patients with respiratory distress most often present [14,15] . As such, doctors working in the EDs need to be familiar with all aspects of NIV usage [2]. Due to the limited availability of specialist emergency medicine physicians in South Africa, the task of initiating NIV will inevitably have to be performed by inexperienced junior doctors [16,17]. Very often, these junior doctors will have had minimal exposure to NIV upon completing their internship, as there is no compulsory Intensive Care Unit (ICU) rotation as a junior doctor and ED exposure is limited to a month rotation [18]. This limited exposure to emergency medicine and critical care suggests that doctors complete their internship without having sufficient experience or training in the use of NIV.

It has been shown that a doctor's prior experience and training in NIV has a direct relationship to their knowledge of NIV [14,[19], [20], [21], [22], [23], [24], [25], [26]]. If NIV is applied with insufficient knowledge and without appropriate training, serious adverse consequences can result [1,20]. Junior doctors who are likely to have little experience with NIV, would therefore demonstrate a lack of knowledge and confidence when initiating NIV [27,28]. This could lead to delays or even avoidance of NIV usage, thereby losing the benefits that this time-sensitive intervention can provide.

Assessing and improving the knowledge and therefore increasing confidence in NIV usage is crucial for all doctors working in emergency departments. Previous studies assessing NIV knowledge and usage in EDs were largely undertaken in European based health centres with no similar South African studies found. Therefore, the aim of this study was to assess the NIV related knowledge, attitudes and practices of emergency doctors in a South African academic setting.

## Methods

This was a multi-centre prospective cross-sectional study. The study was approved by the University of the Witwatersrand Human Research Ethics Committee (M210539). Informed consent was received from all participants who completed the questionnaire.

### Population

The study population at the time of data collection (November 2021 – June 2022) consisted of 128 doctors working in three academic EDs in Johannesburg and in the Division of Emergency Medicine at the University of the Witwatersrand. The three departments are staffed with a total of fourteen emergency medicine specialists and serve a population of approximately 9.2 million [29,30].

The study population was first partitioned by job designation which corresponded to their level of experience. This is represented by using the number of post graduate years (PGY) they have worked in clinical practice. This resulted in the following breakdown: community service doctors (PGY-3), part-time and full-time medical officers (≥PGY-4), emergency medicine registrars (≥PGY-5) and emergency medicine consultants (≥PGY-7).

Doctors completing their internship were not included in the study given that their training as independent medical practitioners would not yet be complete.

### Data collection

A convenience sample of the study population completed the study questionnaire.

The framework for the study questionnaire was adapted from the research conducted by Cabana et al. [31]. Our questionnaire contained 14 knowledge related questions that were adapted from the online accredited Medmastery Non-invasive Ventilation course [32] as well as from the British Thoracic Society (BTS) Guidelines on NIV in acute respiratory failure [1]. NIV was defined as positive pressure ventilatory support delivered by a mask or similar device. There were eight questions relating to practices. The attitude related questions included eight referring to the use of NIV and three referring to the impact of the COVID-19 pandemic on the participants’ use of NIV.

The questionnaire was piloted amongst doctors outside the study population, with similar demographics, to estimate the time needed to complete the questionnaire as well as to identify and correct any misunderstandings, errors, or ambiguity in the questions, with no major issues identified.

The participants anonymously completed the questionnaire either electronically or as a hard copy. All members of the study population were approached in person during various departmental visits and academic meetings. The responses received became the resultant study sample. No incomplete questionnaires were received. The questionnaires were submitted separately from any linking identifiers to maintain participant anonymity. The questionnaire responses were collated onto an Excel® spreadsheet. The electronic data was password protected ensuring that the data was only accessible to the researchers and a statistician.

### Statistical analysis

The statistical analysis was completed using GraphPad Prism 9.4.1. The questions relating to attitudes and practices used Likert scale responses. The responses were assigned numerical values from 1 to 5. The higher value denoting stronger agreement with the statement. The knowledge section included questions which contained multiple correct answers. The questions were allocated negative markings (one negative point for each incorrect answer) to account for the possibility of a participant indiscriminately choosing all available answers. The final knowledge scores were then converted to a percentage of correct answers after negative marking had been applied. The descriptive data is displayed using frequencies and percentages.

One-way ANOVA and *t*-test methods were used to compare groups. Pearson correlation was used to correlate parametric data while Spearman correlation was used to correlate non-parametric data. A p-value of less than 0.05 was considered statistically significant.

## Results

### Demographics

**Fig. 1:**
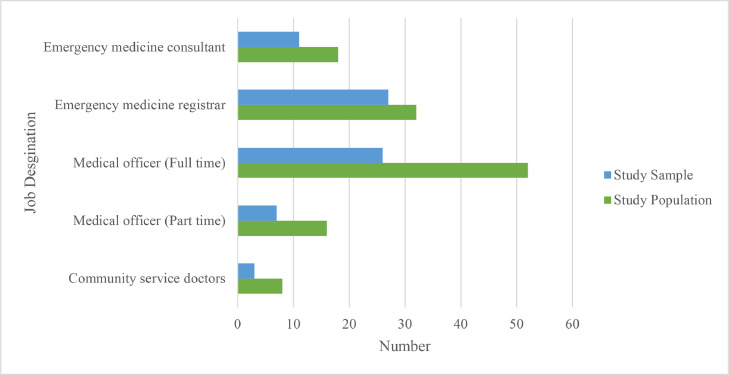
Clustered bar graph displaying the number of participants, partitioned by their job designation, in the study sample relative to the population.

A total of 74 responses were received. The mean age of the study participants was 33.9 years (SD = 6.4). More than half of the participants (57 %) had practiced for 6–10 years and 64 % of participants had worked for 5 years or less in the ED. The sample of participants, once partitioned into their job designation, represented a similar distribution to that of the population Fig. 1.

The training reported by the participants is displayed in Table 1. Of note, 43 % of the participants had received formal NIV training and only 6 % of those did so as a junior doctor (Intern or Community Service Doctor).

**Table 1:** Participant Training Experience.

Formal NIV Training (*N* = 74)	n (%)
Yes	32 (43)
No	42 (57)
Capacity when formal training received (*N* = 32)	
Internship	0 (0)
Community service	2 (6)
Medical officer	18 (56)
Registrar	12 (38)
Consultant	0 (0)
Informal NIV Training (*N* = 74)	
Yes	68 (92)
No	6 (8)
Capacity when informal training received (*N* = 68)	
Internship	3 (4)
Community service	8 (12)
Medical officer	38 (56)
Registrar	17 (25)
Consultant	2 (3)

### Knowledge

**Fig. 2:**
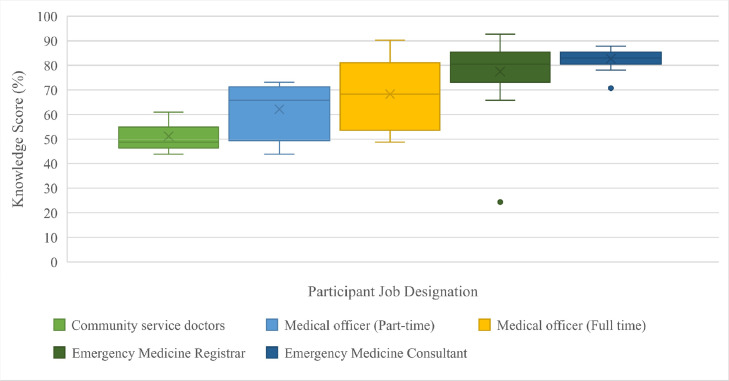
Box and whisker plot representing the participants’ knowledge scores according to their job designation. The ends of the box represent the upper and lower quartiles of the data set. The horizontal line inside the box represents the median. The cross represents the mean. The outer horizontal lines of the whiskers represent the highest and lowest values and the dots represent outliers.

The knowledge scores obtained from the questionnaire demonstrated a strong overall understanding of NIV and its application. The mean knowledge score percentage was 73 % (SD = 13.9). The knowledge of participants was greater if they had more years of experience working in the ED (*p* = 0.001). Fig. 2 displays the knowledge scores according to the participants job designation. There was a statistically significant difference in the knowledge of the participants in different job roles (*p* < 0.001).

The mean knowledge scores were greater in the participants that reported having had critical care experience versus those without (mean 77.3% vs 69 %, *p* = 0.009). This was also present when comparing those with formal NIV training versus those without (77.67% vs 69.5 %, *p* = 0.010). Those that reported having received informal training had a greater mean score compared to those without informal training (73.7% vs 65.9 %, *p* = 0.290). However, this was not statistically significant.

Although the overall knowledge of the participants had a high average score, there were areas of deficiency noted. Regarding indications for NIV, just over a third of participants (36 %) responded that NIV has been shown to have benefit in severe life-threatening hypoxaemia. Only 41 % correctly identified “hypercapnic respiratory failure due to neuromuscular disease” and 55 % correctly selected “weaning from tracheal intubation” as an indication for the use of NIV.

Most participants correctly selected “cardiogenic pulmonary oedema” (93 %) and “COPD with respiratory acidosis” (87 %) as conditions that benefit from the application of NIV.

The question pertaining to contra-indications to the use of NIV demonstrated that the correct contra-indications of “bowel obstruction” and “excessive respiratory secretions” were only selected by 45 % and 43 % of respondents respectively. The other correct contra-indications of “recent facial trauma” and “vomiting” were selected by 95 % and 77 % respectively.

The responses relating to ventilator settings showed that nearly half of the participants (49 %) would change the respiratory rate to improve hypercapnia, which is not possible in a spontaneously breathing patient. Furthermore, 23 % of participants responded that Positive End Expiratory Pressure (PEEP) should be altered to improve hypercapnia. The participants displayed stronger knowledge when correcting for hypoxaemia. However, 18 % would have incorrectly altered pressure support to improve oxygenation.

The question relating to complications from the use of NIV was answered correctly for the most part. However, only 59 % correctly identified “pneumothorax” and 62 % identified “hypotension if hypovolaemic” as complications.

### Attitudes

**Fig. 3:**
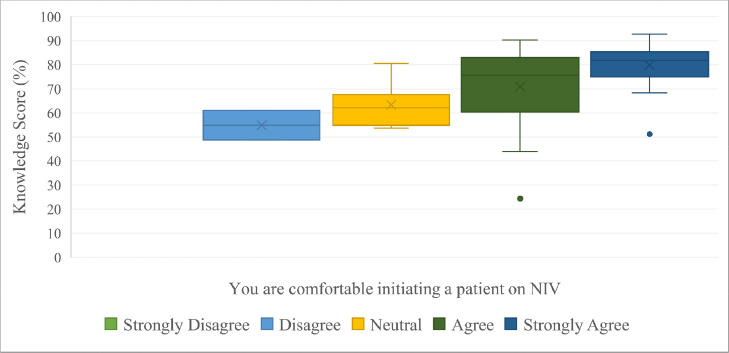
A box and whisker plot displaying the participants’ reported level of comfort when initiating a patient on NIV in relation to their knowledge scores. The ends of the box represent the upper and lower quartiles of the data set. The horizontal line inside the box represents the median. The cross represents the mean. The outer horizontal lines of the whiskers represent the highest and lowest values and the dots represent outliers.

The doctors’ attitudes towards the use of NIV were largely positive. The total possible affirmative Likert score, that could be obtained from all questions pertaining to attitudes, was 40. The mean response was 34 (SD = 3.6). There was a positive correlation between a participant's overall attitude to NIV and their knowledge score (*r* = 0.43, CI [0.22 – 0.60], *p* < 0.001). Fig. 3 graphically demonstrates that participants who had stronger agreement to feeling “comfortable initiating a patient on NIV” had higher mean knowledge scores (*p* < 0.001).

Those that reported to have more frequently avoided or delayed using NIV had lower mean knowledge scores. However, this finding was not statistically significant (*p* = 0.399). The common reasons given for having felt uncomfortable when starting a patient on NIV was lack of equipment (41 %), lack of knowledge (27 %), lack of training (23 %).

The participants’ attitude towards NIV with respect to the recent COVID-19 pandemic demonstrated that 42 % agreed and 19 % strongly agreed that the pandemic had increased their confidence in using NIV. Additionally, 42 % agreed and 18 % strongly agreed that they use NIV more regularly due to the pandemic.

### Practices

Only just over a half of the participants (55 %) reported NIV was always available where they were working and 51 % and 54 % reported they often have sufficient equipment and consumables respectively. Only 8 % of participants often used a checklist while only 4 % always used a checklist when initiating a patient on NIV. The majority (69 %) used no checklist when applying NIV.

Furthermore, only 53 % of participants used a guideline when deciding whether to use NIV for a patient (Table 2). The participants were able to give multiple responses when defining the guidelines that they use in practice. Only 26 % reported using a protocol issued by the department in which they were working and 53 % of participants felt the “decision whether to intubate a patient or not before starting NIV” was not necessary.

**Table 2:** Participant practices relating to the use of NIV.

Do you refer to a resource to decide whether to start NIV or not? (N=74)	n (%)
Yes	39 (53)
No	35 (47)
Resource used when initiating a patient on NIV (N=74)	
Protocol from department in which you work	19 (26)
A Google search	9 (12)
Protocol from an International source	9 (12)
Protocol from another South African source	5 (7)
My knowledge/experience	3 (4)
EM based App on Phone	2 (3)
Consultant on cover	1 (1)
Does the decision whether the patient should be intubated or not need to be made before initiation of NIV? (N=74)	
Yes	34 (46)
No	39 (53)
No Response	1 (1)

Of the 47 % of the participants that do not use a guideline when initiating NIV, 91 % agreed and strongly agreed that they were comfortable initiating NIV on a patient with none of the participants disagreeing or strongly disagreeing with the statement. This contrasts with those that do use a guideline where 11 % disagreed and 8 % responded neutrally, with regards to the statement “are you comfortable initiating NIV”.

Within the 39 participants that do use a guideline, 62 % had not received formal training and of the 35 participants that do not use a guideline, 51 % reported they had not received formal training.

## Discussion

The correct application of NIV is paramount in ensuring positive patient outcomes and safety [1] Therefore, it is imperative that doctors administering NIV are knowledgeable and confident in its use. Previous studies have shown that increased knowledge would lead to increased confidence in the use of NIV [22,23,25]. This finding was reproduced in our study as participants that were more confident using NIV had statistically significant higher average knowledge scores.

The COVID-19 pandemic was shown to have led to increased confidence in the use of NIV by the participants as well as more frequent use. This correlates with research performed by Jackson et al. [33], which reported that the COVID-19 pandemic led to the increased use of NIV to overcome the increased burden on EDs and ICUs.

The knowledge of NIV use in the participants had a direct relationship to their experience in emergency medicine. This was in keeping with results from a United Kingdom NIV knowledge survey conducted by Ballard et al. [19] that demonstrated increased knowledge scores with increased seniority.

Karim et al. [34] have developed an international consensus on NIV training. Their recommendation was to ‘develop structured, organized NIV education and training programs, especially for the developing countries.’ [p. 37] Our study supports this recommendation as knowledge was stronger in those that had received formal NIV training or had past critical care experience. There is a paucity of research relating to how best to train providers in the use of NIV [34]. Our research has helped to highlight areas of deficiency in NIV knowledge.

The participants’ responses demonstrated areas in need of development. These included patient selection, indications, contra-indications, complications, and ventilator settings relating to the use of NIV. Formal training programs should ensure these areas are covered sufficiently and are understood by the trainees. Recommendations for training programs have been made by the BTS as well as the international consensus published by Karim et al. [1,34]. In the South African setting, training programs should follow evidence-based recommendations. This study aids in identifying present weaknesses in the doctors’ knowledge that requires focused attention.

A clear lack of formal and informal training on NIV when working as a junior doctor was demonstrated, highlighting the need for targeting doctors in their earlier (PGY-1 to PGY-3) years when considering a training program in the South African environment. A similar recommendation was made by Mahmud et al. after conducting a UK based NIV survey [23]. This will ensure that doctors are adequately trained in the use of NIV when practicing independently.

Not referring to a resource or protocol when using NIV by 47 % of the participants may have been due to their higher reported level of comfort when initiating NIV. However, our study noted infrequent use of locally developed NIV protocols, as recommended by the BTS guidelines [1]. Therefore, a formal protocol should be promoted in EDs to ensure doctors are following locally applicable NIV procedures. One possible way to improve the implementation of a protocol in the emergency setting would be the development of a NIV checklist, like those developed for invasive ventilation [35].

There is no evidence-based recommendation for the use of a checklist when initiating a patient on NIV and 69 % of this sample of doctors did not report using a checklist. However, within the recommended protocol laid out by the BTS [1], there is a step wise approach to initiating NIV, like that of a checklist. Of note, the first recommendation suggests that before initiating a patient on NIV the clinician must decide and document if the patient will be a candidate for intubation if the NIV management should fail. The practice of deciding whether a patient should be intubated or not, before starting NIV, is practiced by only 46 % of this sample of doctors. This finding may be due to ambiguity of the question or it may be that the recommended practice is not enforced or incorporated into local protocols. This could easily be integrated into a local protocol to improve the practice of having an upfront airway management plan, which would give more junior doctors clearer guidance and align practice with evidence-based standards.

### Study limitations

Although the sample size included more than half (58 %) of the population, the population itself was small and selected from academic centres. This may have created a bias in the results as the baseline knowledge, training and experience may be greater than that of the average emergency doctor in South Africa. Further studies involving a larger sample size and more varied facilities would be warranted to validate the above results.

The types of training and whether it was initial or refresher training was not clearly categorized. The questionnaire did not detail the types of critical care experience, formal and informal training.

NIV was not clearly defined in the questionnaire. Our study refers to NIV delivered by a mask or similar device, but high flow nasal cannula (HFNC) may also be defined as NIV. Participants may have considered the use of HFNC when answering certain questions especially since increasing use of HFNC due to the COVID-19 pandemic [36].

It was not possible to determine if the increase in the use of NIV due to the COVID-19 pandemic was born of necessity or due to an increase in knowledge and confidence in its use.

Convenience sampling and face-to-face recruitment may have increased the risk of response bias as participants with greater knowledge may have been more likely to participate. Furthermore, participants were not made aware that the survey would be assessed with negative marking. It is possible that if they had been aware, their responses may have been more limited.

## Conclusion

The correct use of NIV is essential in ensuring positive patient outcomes. The prior experience, seniority and training of the participant were significant factors influencing their knowledge of NIV use.

Formal NIV training programs aimed at junior doctors, development of a checklist and the implementation of locally developed protocols are recommended to improve the knowledge and confidence of emergency doctors when administering NIV. This research could be used in support of a review into current NIV protocols and training by incorporating the knowledge gaps identified.

## Dissemination of results

Results from this study were shared with the Emergency Medicine Department at the University of the Witwatersrand. The findings may be presented at local, national or international meetings in the future.

## Declaration of Competing Interest

The authors declared no conflicts of interest.
